# Predictive Value of Midsagittal Tissue Bridges on Functional Recovery After Spinal Cord Injury

**DOI:** 10.1177/1545968320971787

**Published:** 2020-11-16

**Authors:** Dario Pfyffer, Kevin Vallotton, Armin Curt, Patrick Freund

**Affiliations:** 1 Balgrist University Hospital, University of Zurich, Zurich, Switzerland; 2 Wellcome Trust Center for Neuroimaging, UCL Institute of Neurology, University College London, London, UK; 3 Max Planck Institute for Human Cognitive and Brain Sciences, Leipzig, Germany

**Keywords:** spinal cord injury, magnetic resonance imaging, recovery of function, decision trees, prognosis

## Abstract

**Background:**

The majority of patients with spinal cord injury (SCI) have anatomically incomplete lesions and present with preserved tissue bridges, yet their outcomes vary.

**Objective:**

To assess the predictive value of the anatomical location (ventral/dorsal) and width of preserved midsagittal tissue bridges for American Spinal Injury Association (ASIA) Impairment Scale (AIS) grade conversion and SCI patient stratification into recovery-specific subgroups.

**Methods:**

This retrospective longitudinal study includes 70 patients (56 men, age: 52.36 ± 18.58 years) with subacute (ie, 1 month) SCI (45 tetraplegics, 25 paraplegics), 1-month neuroimaging data, and 1-month and 12-month clinical data. One-month midsagittal T2-weighted scans were used to determine the location and width of tissue bridges. Their associations with functional outcomes were assessed using partial correlation and unbiased recursive partitioning conditional inference tree (URP-CTREE).

**Results:**

Fifty-seven (81.4%) of 70 patients had tissue bridges (2.53 ± 2.04 mm) at 1-month post-SCI. Larger ventral (*P* = .001, *r* = 0.511) and dorsal (*P* < .001, *r* = 0.546) tissue bridges were associated with higher AIS conversion rates 12 months post-SCI (n = 39). URP-CTREE analysis identified 1-month ventral tissue bridges as predictors of 12-month total motor scores (0.4 mm cutoff, *P* = .008), recovery of upper extremity motor scores at 12 months (1.82 mm cutoff, *P* = .002), 12-month pin-prick scores (1.4 mm cutoff, *P* = .018), and dorsal tissue bridges at 1 month as predictors of 12-month Spinal Cord Independence Measure scores (0.5 mm cutoff, *P* = .003).

**Conclusions:**

Midsagittal tissue bridges add predictive value to baseline clinical measures for post-SCI recovery. Based on tissue bridges’ width, patients can be classified into subgroups of clinical recovery profiles. Midsagittal tissue bridges provide means to optimize patient stratification in clinical trials.

## Introduction

Spinal cord injury (SCI) presents as a clinically manifold disorder with varying degrees of motor, sensory, and autonomic dysfunctions; its magnitude depending on the severity and level of the injury.^1^ Functional recovery is limited^2^ due to permanent damage to neural tissue at the lesion epicenter^3-6^ and progressive secondary neurodegeneration which eventually propagates across the entire neuraxis.^7-9^

Prediction of individual recovery trajectories is of high relevance for patients and for rehabilitation procedure planning.^10-12^ The International Standards for the Neurological Classification of SCI (ISNCSCI)–derived American Spinal Injury Association (ASIA) Impairment Scale (AIS) grade is used to characterize the initial severity of the injury.^13^ Post-SCI recovery is usually clinically observed as an improvement in the AIS grade; currently the best outcome predictor in the very acute phase after the injury.^14-17^ Although the ISNCSCI classification is able to categorize the level of impairment, it can neither fully account for the neurological heterogeneity of SCI patients nor reveal the underlying pathophysiological changes that affect the individual spontaneous recovery trajectories.^18^ To further improve the planning of future clinical trials and post-SCI treatment-tailoring, there is a need to implement alternative objective readouts reflecting changes in the destined therapy targets underlying the patients’ functional status.^19^

Neuroimaging biomarkers based on intramedullary structural changes have been identified using conventional magnetic resonance imaging (MRI), and these complement the diagnostic workup and outcome prediction after SCI.^6,8,20^ Particularly, the degree of recovery after SCI can be predicted by MRI readouts at the lesion level.^21-25^ At the subacute stage after SCI, preserved midsagittal tissue bridges adjacent to the intramedullary cystic cavity and the spinal canal were shown to be permissive for electrophysiological information flow and associated with neurological and functional recovery.^3-5^ It remains uncertain whether the anatomical location and width of preserved tissue bridges are reliable predictors of AIS grade conversion and improve the stratification of SCI patients into distinct recovery subgroups. Unbiased recursive partitioning conditional inference tree (URP-CTREE)^26^ analysis has proven a powerful tool for prediction-based stratification of patients with complete^27^ and incomplete^28^ SCI, classifying patients into more homogeneous recovery subgroups. This study therefore investigated the potential role of ventral and dorsal tissue bridges in predicting AIS grade conversion as well as recovery of sensorimotor function and daily life independence after SCI. It further aimed to identify the predictive value of tissue bridges as covariates in prediction-based stratification analyses.^16,17^

## Methods

### Experimental Design

Seventy patients with subacute SCI (45 tetraplegic and 25 paraplegic patients) were included in this retrospective study (Table 1). These patients were admitted consecutively to the Balgrist University Hospital (Zurich, Switzerland) between May 2002 and March 2019.

**Table 1.** Demographical, Clinical, and Structural Neuroimaging Data of All Study Participants.

PID	Age, y	AIS grade at 1 mo/12 mo	NLI at 1 mo/12 mo	Presence of v/d tb at 1 mo	PID	Age, y	AIS grade at 1 mo/12 mo	NLI at 1 mo/12 mo	Presence of v/d tb at 1 mo
1	27	A/A	C3/C4	n/n	36	21	D/D	C3/C8	y/y
2	17	A/A	C4/C6	y/y	37	59	D/D	C3/C3	y/y
3	30	A/A	C4/C4	n/n	38	63	D/D	C3/C7	y/y
4	19	A/B	C5/C5	n/y	39	67	D/D	C3/L3	y/y
5	32	A/A	C6/C7	n/n	40	47	D/D	C3/C4	y/y
6	41	A/A	C6/C4	n/n	41	75	D/D	C4/C7	y/y
7	30	A/A	T8/T8	n/n	42	62	D/D	C4/C5	y/y
8	29	A/A	T10/T10	n/n	43	66	D/D	C4/C3	y/y
9	77	A/A	T10/T10	n/n	44	47	D/D	C4/C1	y/y
10	66	A/A	T11/T12	n/n	45	23	D/D	C4/T4	y/y
11	71	A/A	T11/T11	n/n	46	58	D/D	C4/C5	y/y
12	72	A/A	T11/T11	n/n	47	53	D/E	C4/int	y/y
13	83	A/D	L1/L2	n/n	48	65	D/E	C4/int	y/y
14	29	B/C	C3/C2	y/n	49	61	D/D	C4/C4	y/n
15	51	B/C	C5/C5	y/y	50	60	D/D	C4/C2	y/y
16	55	B/B	C5/C5	n/n	51	58	D/D	C4/C4	y/y
17	41	B/D	C6/C5	y/y	52	36	D/D	C4/T5	y/y
18	37	B/B	C6/C7	y/y	53	65	D/D	C5/C5	y/y
19	30	B/B	C7/C7	n/y	54	52	D/D	C5/C5	y/y
20	67	B/D	T9/T11	y/y	55	52	D/D	C5/C5	y/y
21	65	C/C	C3/C4	y/y	56	61	D/D	C7/C4	y/y
22	66	C/D	C3/C2	y/n	57	32	D/D	C7/T5	y/y
23	22	C/D	C4/C6	y/y	58	57	D/D	T3/T4	n/y
24	53	C/D	C4/C3	y/n	59	71	D/E	T4/int	y/y
25	30	C/D	C6/C5	y/y	60	38	D/D	T6/T6	y/n
26	70	C/C	C7/T1	n/y	61	70	D/D	T7/L3	n/y
27	80	C/D	T3/T3	y/n	62	27	D/D	T7/T9	y/y
28	31	C/D	T4/T8	n/y	63	44	D/D	T9/T9	n/y
29	85	C/C	T8/T7	n/y	64	59	D/D	T10/T11	y/y
30	71	C/D	T10/T10	y/n	65	53	D/D	T10/T12	y/n
31	73	C/D	T12/L1	y/n	66	77	D/D	T10/T11	y/y
32	68	D/D	C1/T1	y/y	67	46	D/D	T11/T12	y/y
33	72	D/D	C2/C5	y/y	68	29	D/D	T11/T11	n/n
34	53	D/D	C2/C4	y/n	69	79	D/D	T12/T12	y/y
35	65	D/D	C3/C3	y/n	70	24	D/D	L2/L3	y/y

Abbreviations: AIS, American Spinal Injury Association Impairment Scale; d, dorsal; int, intact; NLI, neurological level of injury; PID, participant identifier; tb, tissue bridges; v, ventral.

Every patient had a baseline (ie, 1 month) neuroimaging and clinical assessment as well as a follow-up clinical assessment at 12 months after the injury. These data were used to investigate clinicopathological relationships and to infer on clinical outcomes based on early neuroimaging parameters and clinical measures using regression analyses in terms of partial correlation analysis and unbiased recursive partitioning (URP).^26^

SCI patients with preexisting neurologic or mental disorders or brain lesions were excluded. We furthermore excluded patients with a lumbosacral injury (ie, cauda equina syndrome) as well as patients with MRI contraindications.

The local ethics committee approved the study protocol (EK-2010-0271), which was conducted in accordance with the Declaration of Helsinki. All patients with SCI gave informed, written consent prior to study enrollment. Tissue bridge data from a subset of the study population was previously reported in a different context.^3-5^

### Clinical Assessments

Patients with SCI underwent a comprehensive clinical examination protocol at 1 and 12 months, including (1) the International Standards for the Neurological Classification of Spinal Cord Injury (ISNCSCI) protocol^13^ and (2) the Spinal Cord Independence Measure (SCIM) questionnaire to assess everyday performance and disability. The ISNCSCI protocol included the total motor score (the sum of upper and lower extremity motor scores) of 10 key muscle groups on both sides of the body, as well as the pin-prick and light-touch score of 28 dermatomes on each side of the body. According to these scores, the neurological level of injury and the lesion severity in terms of the AIS grade (i.e. AIS A, complete lesion; AIS B-D, incomplete lesion; AIS E, no functional impairment) will be determined. The normalized AIS conversion rate from 1 to 12 months was calculated by dividing the number of AIS grades actually improved by the maximum number of AIS grades potentially improvable. ISNCSCI data were available for all patients at 1 and 12 months post-SCI. The SCIM questionnaire consists of 3 different subparts with questions regarding self-care, respiration and sphincter management, and mobility. Data from that questionnaire were available for 65 (42 tetraplegic and 23 paraplegic patients) and 68 (45 tetraplegic and 23 paraplegic patients) patients at 1 and 12 months post-SCI, respectively.

### Image Acquisition

All patients with SCI in this study were scanned at 1.5-T or 3-T on Philips (Philips Healthcare), Siemens (Siemens Healthcare), or GE (GE Medical Systems) MRI scanners. All scanners had a 32-channel receive spine coil integrated in the table. The anatomical MRI protocol consisted of conventional sagittal T1-weighted (T1w), sagittal T2-weighted (T2w), and axial T2w clinical scans obtained at the lesion level. For lesion segmentation analysis, only the midsagittal slice of all sagittal T2w images was used in every patient.^3-5^

### Image Analysis

Already at the early stage after SCI, intramedullary damage manifests as changes of signal intensity on T2w scans.^29^ However, neural damage and the resulting cerebrospinal fluid (CSF)–filled cystic cavity is hardly distinguishable from edema at the acute stage, as edema is not fully resolved at that time and both lead to hyperintense signal changes in the spinal cord. Crucially, at the subacute stage, edema has largely resolved and the well-demarcated hyperintense region likely reflects intramedullary neural damage rather than edema.^3,5^ Patients’ T2w scans were only included if the lesion was clearly visible (ie, distinct hyperintense signal) on the midsagittal slice. Images with metal artifact-induced insufficient image quality or lesion visibility were excluded.^3-5^ Neuroimaging data with appropriate image quality and lesion visibility at baseline (i.e. sub-acute stage) was available for all 70 patients with SCI.

**Figure 1.**
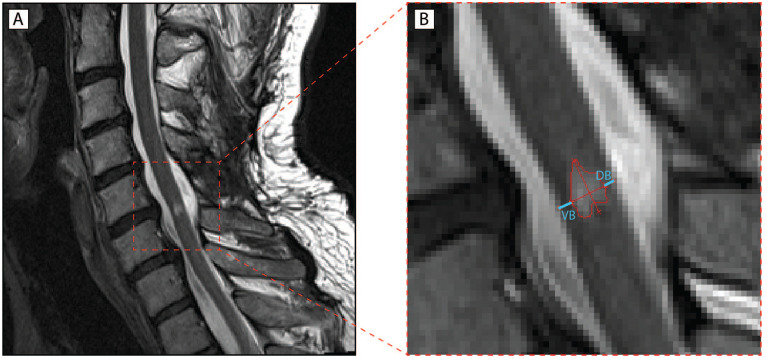
Quantitative assessment of midsagittal tissue bridges. (A) Representative T2-weighted midsagittal image of an intramedullary cystic cavity formed after spinal cord injury (SCI). (B) Schematic lesion segmentation including ventral tissue bridges (VB) and dorsal tissue bridges (DB) highlighted in light blue. Identification and quantification of magnetic resonance imaging (MRI) measures is performed manually in Jim. Tissue bridges are defined as the relatively hypointense region between the hyperintense cystic cavity and the spinal canal.

Lesion segmentation was performed manually at the injury site with the Jim software (version 7.0, Xinapse Systems) and the rater (DP) was blinded to patient identity. In Jim, the lesion was identified on the midsagittal slice from sagittal T2w scans. Assessment of lesion parameters included lesion area, rostro-caudal lesion length, anterior-posterior lesion width, and ventral and dorsal tissue bridges, both together summing up to the total width of tissue bridges. On that midsagittal slice, tissue bridges were defined as those low-signal regions located next to the relatively hyperintense intramedullary cystic cavity and the spinal canal filled with CSF.^3,5^ Ventral and dorsal tissue bridges’ width was quantified as the shortest distance between the cystic cavity and the ventral and dorsal edge of the spinal canal, respectively, at an angle of 90° to the rostro-caudal alignment of the spinal cord (Figure 1).

### Statistical Analysis

We used Stata (version 14.2; StataCorp LP) and R (version 3.4.3) software to perform the statistical analysis of these study data.

Nonparametric tests were used for group comparisons to account for the nonnormally distributed data. Kruskal-Wallis tests were applied to check for differences in 1-month ventral and dorsal tissue bridges across SCI patient subgroups with different AIS grades at 12 months post-SCI. Moreover, we used Mann-Whitney *U* tests to compare patients who improved in AIS grade from the acute and subacute to the chronic stage to patients who did not show AIS grade conversion over time, regarding their 1-month ventral and dorsal tissue bridges’ width. We also applied Mann-Whitney *U* tests to compare 1-month widths of ventral and dorsal tissue bridges in patients who improved their AIS grade to C or D at 12 months post-SCI. Additionally, patients with both ventral and dorsal tissue bridges were compared to patients with ventral tissue bridges only and patients with dorsal tissue bridges only at 1 month, regarding their 12-month SCIM score.

Partial correlation analysis was applied to explore potential relationships between imaging parameters (ie, midsagittal ventral and dorsal tissue bridges) and AIS grades at 12 months post-injury as well as normalized AIS conversion rates from 1- to 12-months (i.e. actual number of AIS grades improved divided by maximum number improvable). In these regression models, age, sex, and ventral or dorsal tissue bridges, respectively, were included as covariates of no interest to adjust for their dependency. Potential confounders of the partial correlation analysis were only kept in the model if the covariates of no interest were significant or had a considerable effect on the correlation coefficient. Results with a *P* value ≤.05 were regarded as significant.

In both group comparison and regression analyses including AIS conversion rates, only SCI patients with AIS grades A to C at the acute stage (n = 39) or at 1 month (n = 31) were considered to exclude patients with a very low AIS conversion chance (ie. patients with AIS grade D) and therefore avoid roof effects.

To account for the neurological heterogeneity within the SCI population and identify patient subgroups based on their functional recovery, we then applied an unbiased recursive partitioning technique^27,28,30-32^ called URP-CTREE^26^ implemented in the “party” package within R (version 3.4.3).^33^ A detailed description of the method and how its algorithm works can be found in Hothorn and Zeileis.^26^ In short, URP-CTREE is a regression model that uses a set of predictors (eg, neuroimaging parameters like tissue bridges at 1 month) and independently tests them to prognosticate future clinical endpoints (eg, total motor score at 12 months). Presented as a tree-structured model, it performs a prospective prediction-based stratification of an initial heterogeneous patient cohort into more homogeneous subgroups with regard to predefined clinical outcomes. The algorithm in the URP-CTREE model works in a way that it dichotomously separates the initial patient cohort stepwise into well-defined pairs of subgroups (ie, nodes) with regard to the clinical endpoint defined. If the patients of such a node will be further separated, it is called an inner node. The algorithm continues splitting as long as one of the predictor variables is found to separate this subgroup into 2 more distinct subgroups with a *P* value ≤.05. In that, it is programmed to identify the singular most significant predictor for every separation step and to go on as long as it finds a significant predictor with its corresponding cutoff value. Therefore, it aims to ultimately maximize the difference between all newly formed subgroups of the outer nodes (eg, indicated by box plots at the bottom of the tree).

In our study, we used (1) ventral and dorsal midsagittal tissue bridges, total motor score, age, and lesion level at 1 month as predictors and 12-month total motor score as the clinical endpoint; (2) ventral and dorsal midsagittal tissue bridges, upper extremity motor score (UEMS), and age at 1 month as predictors and the change in UEMS from 1 to 12 months as the clinical endpoint; (3) ventral and dorsal midsagittal tissue bridges, pin-prick score, age, and lesion level at 1 month as predictors and 12-month pin-prick score as the clinical endpoint; and (4) ventral and dorsal midsagittal tissue bridges, acute AIS grade, age, and lesion level at 1 month as predictors and 12-month SCIM score as the clinical endpoint. Baseline SCIM score was not used as a predictor here as it is not a highly reliable measure for functional independency at this stage due to possible comorbidities and other constraints.

### Data Availability

On request, anonymized data of this study are available from the corresponding author.

## Results

### Demographics and Neuroimaging Readouts

This study includes 70 patients with SCI (n = 56 men, 80.0%) with a mean ± standard deviation age of 52.36 ± 18.56 years (Table 1). Their time interval between injury and baseline scan at 1 month (ie, subacute stage) was 40.50 ± 46.86 days. At 1 month, 13 patients had a complete lesion (ie, AIS grade A). Seven patients were graded AIS B, 11 patients AIS C, and 39 patients AIS D. Seventeen (24.3%) of these patients improved in AIS grade over time whereas 53 patients (75.7%) stayed at the same grade (see Table 1 for more detail).

Of all 70 SCI patients with a neuroimaging assessment at 1 month, 57 (2 AIS A patients) had midsagittal tissue bridges (81.4%) with a width of 2.53 ± 2.04 mm while 13 (11 AIS A patients) had no detectable midsagittal tissue bridges (18.6%). Forty-nine patients (70.0%) had ventral tissue bridges at 1 month with a width of 1.36 ± 1.32 mm and 21 patients (30.0%) did not have ventral tissue bridges at baseline. While 46 patients (65.7%) did have dorsal tissue bridges at 1 month with a width of 1.18 ± 1.28 mm, 24 patients (34.3%) did not have any dorsal tissue bridges.

### Midsagittal Tissue Bridges Differentiate Patients With SCI According to Their Future AIS Grade

**Figure 2.**
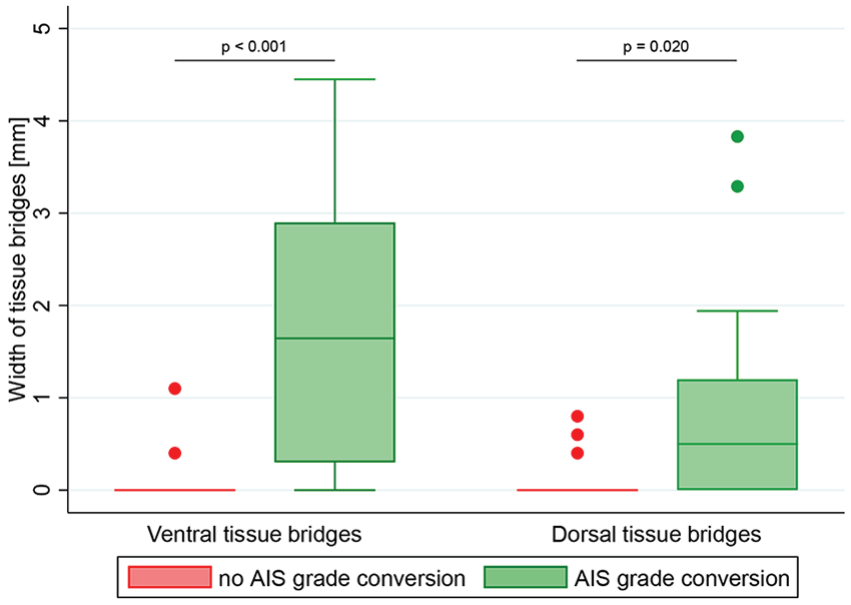
Comparison of tissue bridges between spinal cord injury (SCI) patients with and without American Spinal Injury Association Impairment Scale (AIS) grade conversion. Group differences in ventral and dorsal tissue bridges between SCI patients without (indicated in red) and with (indicated in green) conversion of AIS grade from the acute stage to 12 months post-SCI are shown with box plots and corresponding error bars (ie, whiskers). Patients with a tissue bridges’ width larger than 1.5 times of the interquartile range above the upper quartile (ie outliers) are represented as red (without conversion) and green (with conversion) dots. Significant differences are reported with uncorrected *P* values.

Subgroup comparison of patients with SCI according to their AIS grade at 12 months identified an overall difference in ventral (*P* < .001) and dorsal (*P* = .018) tissue bridges at 1 month. Patients with AIS grades A to C at 1 month (n = 31) who showed AIS grade conversion (n = 13) had significantly larger ventral (*P* = .001) but not dorsal (*P* = .113) tissue bridges when compared to patients without conversion (n = 18). However, when referring to the AIS grade conversion of AIS A to C patients from the early (ie, acute) stage (n = 39) to 12 months post-SCI (Figure 2), both ventral (*P* < .001) and dorsal (*P* = .020) tissue bridges were larger in patients who improved in AIS grade (n = 26) when compared with patients who stayed at the same grade (n = 13). Moreover, patients who improved their acute AIS grade to grade C or D at 12 months (n = 23) had significantly larger ventral (1.64 ± 1.30 mm) than dorsal (0.70 ± 0.93 mm) tissue bridges (*P* = .010).

### Midsagittal Tissue Bridges Are Associated With AIS Conversion Rates

**Figure 3.**
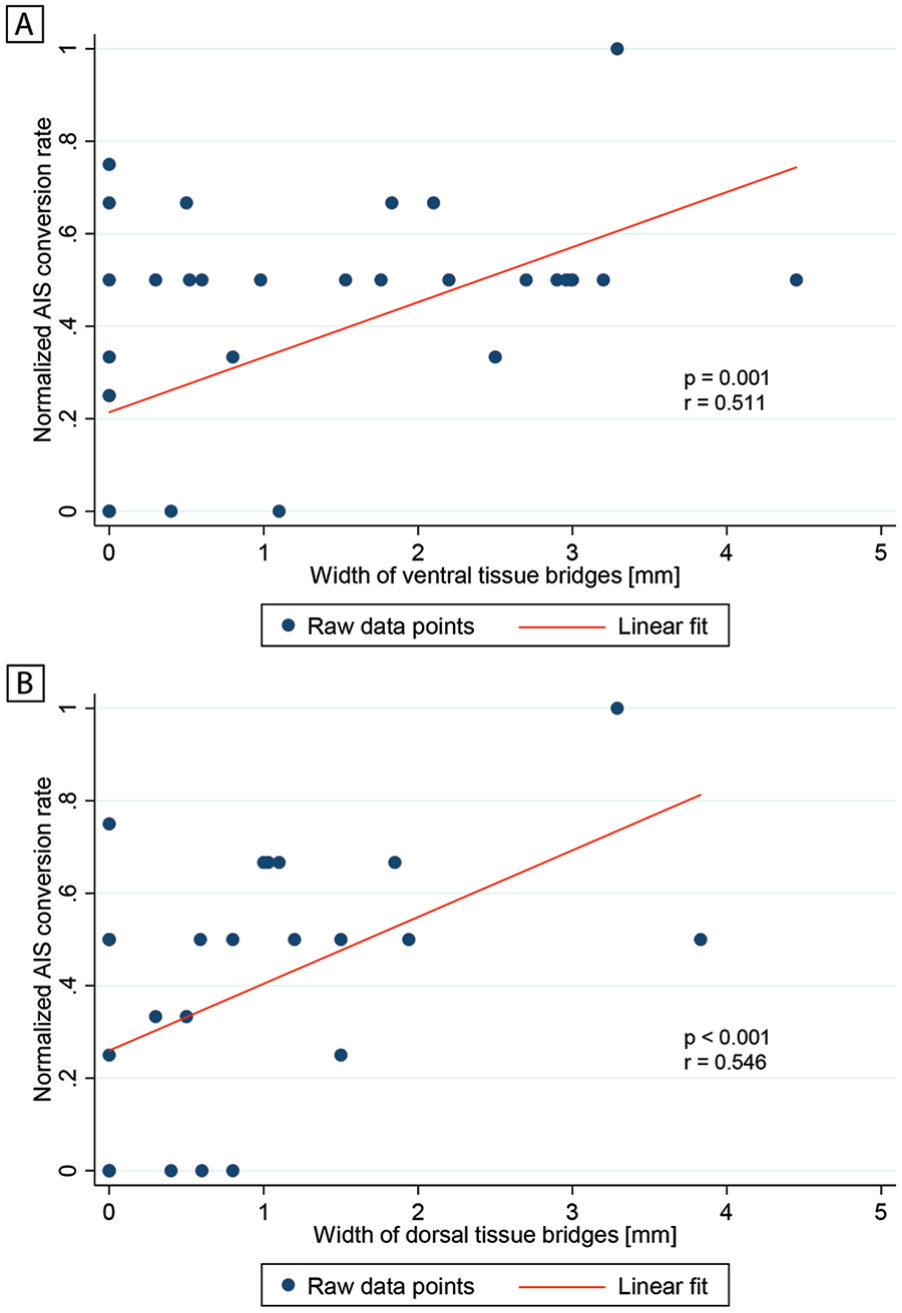
Relationship between normalized American Spinal Injury Association Impairment Scale (AIS) conversion rate and width of tissue bridges. Partial correlation graphs showing the associations between the normalized AIS conversion rate and width of (A) ventral tissue bridges and (B) dorsal tissue bridges. The normalized AIS conversion rate is defined as the actual number of AIS grades improved divided by the maximum number of AIS grades improvable. Raw data points are indicated by blue dots and the linear fit by a red line. Significant associations are reported using uncorrected *P* values and correlation coefficients *r*.

Both larger ventral (*P* < .001, *r* = 0.531, n = 70) and dorsal (*P* < .001, *r* = 0.506, n = 70) tissue bridges were associated with higher AIS grades at 12 months, independent of age, sex, and dorsal or ventral tissue bridges, respectively. In SCI patients with AIS grades A to C at the early stage, ventral tissue bridges were positively correlated with the normalized AIS conversion rate (ie, actual number of AIS grades improved divided by maximum number improvable) 12 months after SCI (*P* = .001, *r* = 0.511, n = 39, Figure 3A), independent of age, sex, and dorsal tissue bridges. Similarly, a larger width of dorsal tissue bridges was correlated with a higher normalized AIS conversion rate 12 months post-SCI (*P* < .001, *r* = 0.546, n = 39, Figure 3B), independent of age, sex, and ventral tissue bridges.

### Midsagittal Tissue Bridges Are Associated With Functional Recovery

**Figure 4.**
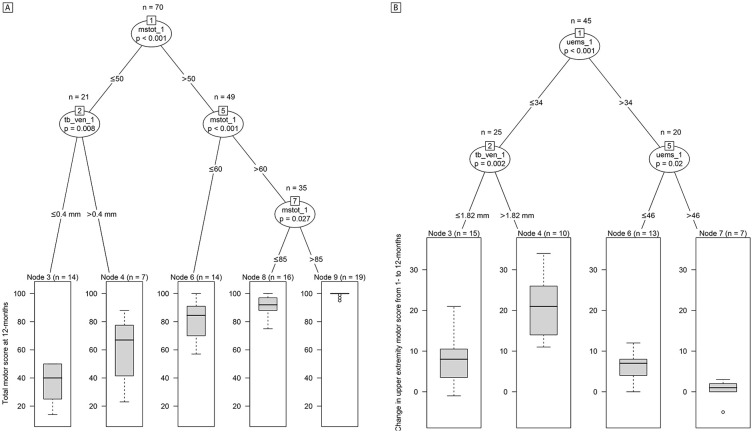
Conditional inference trees for 12-months total motor score and change in upper extremity motor score. Unbiased recursive partitioning conditional inference trees with clinical endpoints of (A) total motor score at 12 months and (B) change in upper extremity motor score from 1 to 12 months. (A) For all spinal cord injury (SCI) patients in the model (n = 70), 1-month ventral and dorsal tissue bridges, 1-month total motor score, age, and lesion level were used as predictors. The algorithm separated the initial patient population (node 1) into 3 inner (nodes 2, 5, and 7) and 5 terminal nodes (nodes 3, 4, 6, 8, and 9). (B) For all tetraplegic SCI patients in the model (n = 45), 1-month ventral and dorsal tissue bridges, 1-month upper extremity motor score, and age were used as predictors. The algorithm separated the initial patient population (node 1) into 2 inner (nodes 2 and 5) and 4 terminal nodes (nodes 3, 4, 6, and 7). The clinical endpoint distribution of the resulting more homogeneous subgroups at the terminal nodes are shown as box plots with 2-sided error bars at the bottom. Significant differences are reported with multiple testing-corrected (ie, Bonferroni-corrected) *P* values. mstot_1, total motor score at 1 month; uems_1, upper extremity motor score at 1 month; tb_ven_1, width of ventral tissue bridges at 1 month.

URP-CTREE analysis separated the entire SCI patient population (n = 70) into 2 nodes (*P* < .001) with regard to the patients’ total motor score at 12 months (Figure 4A), according to their 1-month total motor score being ≤50 (n = 21, node 2) or >50 (n = 49, node 5). In a next step, the algorithm identified width of 1-month ventral tissue bridges as a second predictor variable for node 2 and separated this subgroup into 2 more nodes (*P* = .008) with ≤0.4 mm (n = 14, node 3) and >0.4 mm (n = 7, node 4) in ventral tissue bridges’ width. These 2 subgroups presented 12-month total motor scores of 36.79 ± 13.97 and 59.43 ± 24.27, respectively. Node 5 was furthermore split into nodes 6 and 7 (*P* < .001), according to the 1-month total motor score being ≤60 (n = 14, node 6) or >60 (n = 35, node 7). Finally, the same predictor variable separated node 7 into 2 subgroups (*P* = .027) with a 1-month total motor score of ≤85 (n = 16, node 8) or >85 (n = 19, node 9). The subgroups of nodes 6, 8, and 9 presented 12-month total motor scores of 82.29 ± 13.36, 91.44 ± 7.16, and 99.26 ± 1.66, respectively.

In tetraplegic patients only, URP-CTREE analysis split the population (n = 45) into 2 nodes (*P* < .001) with regard to their change in UEMS from 1 to 12 months (Figure 4B), according to the UEMS at 1 month being ≤34 (n = 25, node 2) or >34 (n = 20, node 5). Node 2 was furthermore separated into nodes 3 and 4 (*P* = .002) with ≤1.82 mm (n = 15, node 3) and >1.82 mm (n = 10, node 4) in 1-month ventral tissue bridges’ width. These 2 subgroups presented a change in UEMS of 7.73 ± 5.56 and 20.50 ± 7.12, respectively. Node 5 was also split once more into 2 subgroups of nodes 6 and 7 (*P* = .020), according to the 1-month UEMS being ≤46 (n = 13, node 6) or >46 (n = 7, node 7). These patients presented a change in UEMS of 6.00 ± 3.54 and 0.43 ± 2.64, respectively, over time.

**Figure 5.**
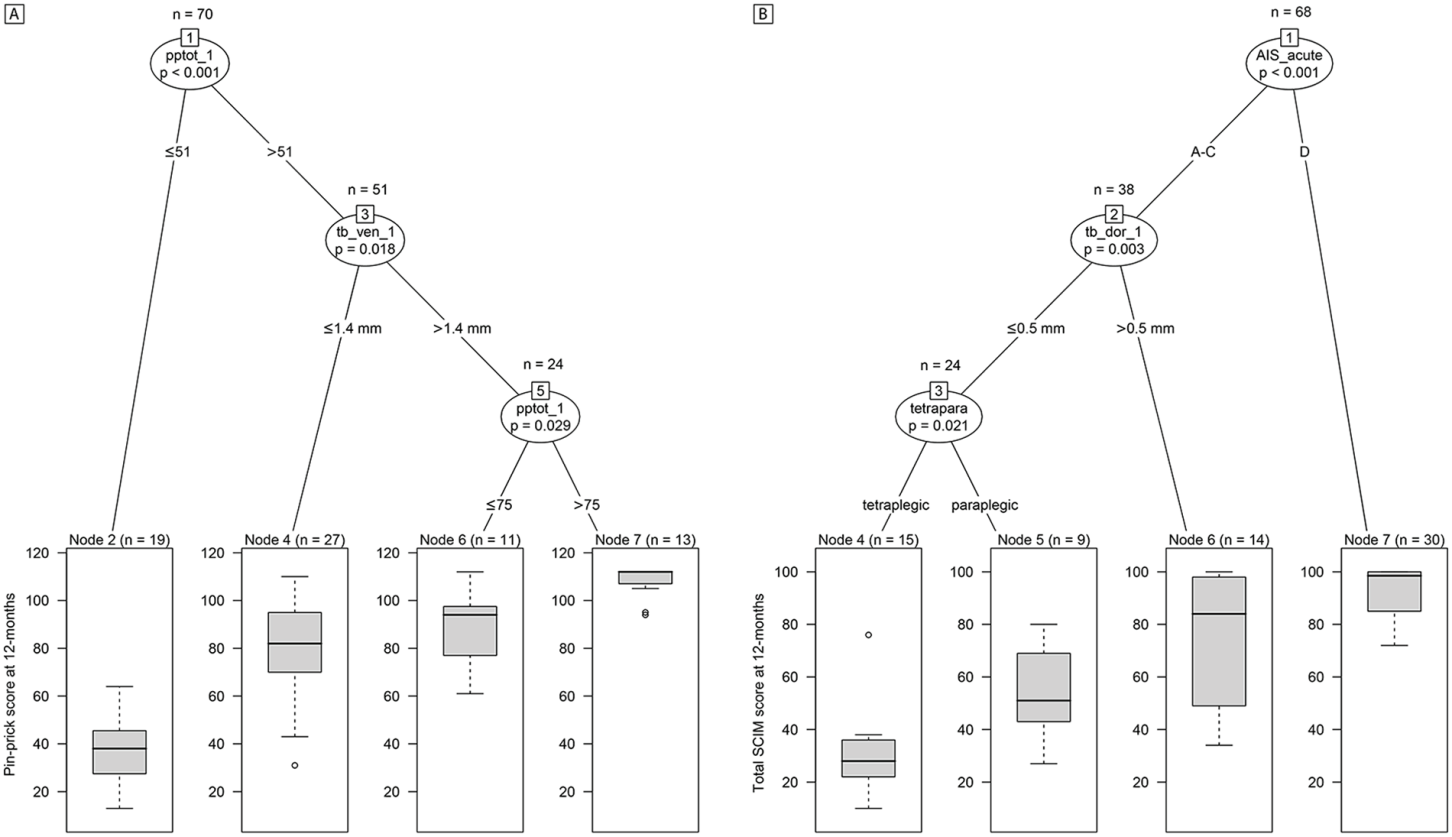
Conditional inference trees for 12-month pin-prick and Spinal Cord Independence Measure (SCIM) score. Unbiased recursive partitioning conditional inference trees with clinical endpoints of (A) pin-prick score at 12 months and (B) total SCIM score at 12 months. (A) For all spinal cord injury (SCI) patients in the model (n = 70), 1-month ventral and dorsal tissue bridges, 1-month pin-prick score, age, and lesion level were used as predictors. The algorithm separated the initial patient population (node 1) into 2 inner (nodes 3 and 5) and 4 terminal nodes (nodes 2, 4, 6, and 7). (B) For all SCI patients in the model with SCIM data available at 12 months (n = 68), 1-month ventral and dorsal tissue bridges, AIS grade at acute stage, age, and lesion level were used as predictors. The algorithm separated the initial patient population (node 1) into 2 inner (nodes 2 and 3) and 4 terminal nodes (nodes 4, 5, 6, and 7). The clinical endpoint distribution of the resulting more homogeneous subgroups at the terminal nodes are shown as box plots with 2-sided error bars at the bottom. Significant differences are reported with multiple testing-corrected (ie, Bonferroni-corrected) *P* values. AIS_acute, American Spinal Injury Association Impairment Scale grade at acute stage; pptot_1, total pin-prick score at 1 month; b_dor_1, width of dorsal tissue bridges at 1 month; tb_ven_1, width of ventral tissue bridges at 1 month; tetrapara, lesion level (ie, tetraplegic or paraplegic).

In terms of sensory recovery, URP-CTREE analysis with 12-month pin-prick score as the clinical endpoint (Figure 5A) led to a partition of the whole patient population (n = 70) into 2 nodes (*P* < .001), according to their pin-prick score at 1 month being ≤51 (n = 19, node 2) and >51 (n = 51, node 3). In a next step, the algorithm identified width of ventral tissue bridges at 1 month as a second predictor variable, separating node 2 patients into 2 more subgroups (*P* = .018) with ≤1.4 mm (n = 27, node 4) and >1.4 mm (n = 24, node 5) in ventral tissue bridges’ width. Node 5 was then split into 2 last nodes (*P* = .029) with a 1-month pin-prick score of ≤75 (n = 11, node 6) or >75 (n = 13, node 7). The 4 resulting subgroups presented 12-month pin-prick scores of 37.53 ± 15.13, 80.37 ± 18.73, 87.45 ± 15.12, and 107.85 ± 6.38, respectively.

With regard to functional independence in daily life activities, URP-CTREE analysis identified the acute AIS grade as a predictor for the SCIM score at 12 months (Figure 5B) and separated the entire patient cohort (n = 68) into 2 subgroups (*P* < .001) with an AIS grade of A to C (n = 38, node 2) or D (n = 30, node 7). The subgroup of node 7 presented a 12-month SCIM score of 91.97 ± 10.62. As a second predictor variable, the algorithm identified dorsal tissue bridges’ width at 1 month, splitting patients of node 2 into 2 more subgroups (*P* = .003) with a width in dorsal tissue bridges of ≤0.5 mm (n = 24, node 3) and >0.5 mm (n = 14, node 6). Node 6 patients presented a SCIM score of 75.79 ± 24.64 at 12 months. In a final step, lesion level (ie, tetraplegic or paraplegic) separated node 3 patients into 2 last subgroups (*P* = .021), tetraplegic patients (n = 15, node 4) and paraplegic patients (n = 9, node 5) presenting 12-month SCIM scores of 30.33 ± 15.14 and 54.56 ± 18.58, respectively. Interestingly, difference in 12-month SCIM scores was significant (*P* = .006) between patients with both ventral and dorsal tissue bridges (n = 37) and patients with ventral tissue bridges only (n = 11) whereas it was trend significant (*P* = .050) between patients with both ventral and dorsal tissue bridges (n = 37) and patients with dorsal tissue bridges only (n = 8).

## Discussion

This study identified preserved ventral and dorsal midsagittal tissue bridges at 1 month post-SCI as predictors of AIS conversion. In particular, ventral tissue bridges were larger in AIS converters when compared to non-converters. Crucially, using URP-CTREE analyses, we identified the prognostic value of ventral tissue bridges for recovery of motor scores and pin-prick scores and dorsal tissue bridges for recovery of functional independence in daily life activities (ie, SCIM score), irrespective of baseline clinical measures. These findings allow us to critically discuss the value of preserved tissue bridges—based on a large SCI cohort—in predicting neurological and functional recovery post-SCI and for prediction-based stratification of patients in clinical trials.

Improvement in AIS grade and recovery of motor, sensory, and functional independence (ie, SCIM) scores is best predicted by baseline AIS grade and clinical scores.^16,34,35^ Recently, Aarabi et al^24^ and Farhadi et al^25^ evaluated the role of MRI biomarkers at the lesion level in predicting post-SCI recovery. They reported that the intramedullary lesion length (IMLL)^24,25^ and the Brain and Spinal Injury Center (BASIC) score^25^ are strong predictors of AIS grade conversion at 6 and 12 months post-SCI, respectively. Moreover, Martineau et al^36^ and Dalkilic et al^37^ showed that presence^36^ or length^37^ of intramedullary hemorrhage, lesion length,^36^ and cord expansion length (ie, entire length of cord that was enlarged as compared with normal)^37^ predicted motor score recovery^36,37^ and AIS grade conversion.^36^ Supported by findings in the literature,^15,38^ we observed that from 70 patients, 28 converted on the ISNCSCI-based impairment scale over 12 months post-SCI with different conversion chances between AIS grades. While patients initially classified as AIS C have the greatest chance to convert, patients with AIS grade B have a lower conversion chance, followed by grade A and then D. Those who converted to an AIS grade of C or D had a larger width of ventral tissue bridges at baseline. This supports the notion that ventral tissue bridges—which likely include spared motor tracts^4,39^—are more preserved in motor incomplete AIS C and D patients.^13^ Dorsal tissue bridges, on the other hand, might rather play a role in conversion from AIS grade A to B, which is less frequently observed in SCI patients.^14,40-43^ In our patient cohort, only 2 patients improved from AIS grade A to B, one of them with only dorsal tissue bridges preserved and the other one without any detectable tissue bridges.

By means of URP-CTREE we show that ventral and dorsal tissue bridges add predictive value to baseline clinical scores for stratification of SCI patients with regard to their functional recovery. Specifically, the algorithm identified 1-month ventral tissue bridges, in addition to baseline motor scores, as predictors of 12-month total motor score and recovery of UEMS. The latter conditional inference tree applies to tetraplegic patients and supports the findings from Velstra et al^32^ regarding prediction of upper limb function. Interestingly, ventral tissue bridges separated the clinically most impaired individuals regarding both 12-month total motor score and recovery of UEMS over time, whereas baseline motor scores were more important for separating out patients with higher motor scores or lower changes in UEMS.

Similarly, the algorithm separated the initial heterogeneous patient cohort into more homogeneous subgroups with regard to their 12-month pin-prick and SCIM scores. Ventral tissue bridges at 1-month were again identified as strong predictors of 12-month pin-prick score. Similar to motor commands, pin-prick information is known to be mediated by predominantly ventrally located spinal cord tracts.^39,44^ URP-CTREE further identified dorsal tissue bridges, in addition to the acute AIS grade and lesion level, as predictors of 12-month SCIM score. Interestingly, dorsal tissue bridges seem to be more important for recovery of functional independence after SCI then ventral tissue bridges, which is also supported by the observed difference in SCIM scores between patient subgroups with regard to their tissue bridges’ anatomical location. It might well be that activities of the daily living, which are covered by the SCIM questionnaire, are more dependent on intact intramedullary tracts included in dorsal rather than ventral tissue bridges. Specifically, the questionnaire covers respiration as well as bowel and bladder function, all of which are regulated by the autonomic nervous system whose tracts run partly dorsally (eg, medial forebrain bundle, dorsal longitudinal fasciculus) within the spinal cord.^45,46^

We also tested the stratification of patients into recovery-specific subgroups with regard to the aforementioned clinical endpoints, including the lesion area as a predictor instead of ventral and dorsal tissue bridges. However, this did not improve the overall accuracy of the URP-CTREE models, either observed by fewer outer nodes (ie, resulting subgroups), increased *P* values of the inner nodes, or the lesion area not showing up as a significant predictor within the tree. Similarly, when just removing ventral and dorsal tissue bridges and sticking to clinical factors as predictors only, the overall accuracy of the URP-CTREE models was lower, reflected by fewer outer nodes and/or increased inner node *P* values. Generally, the choice of the clinical endpoint influences what predictors are significantly related to it. Independent from the number of predictors entered into the model, it will only identify the ones significantly splitting up patients into subgroups and accurately predicting their future clinical outcomes, with a stricter correction for multiple comparison if more predictors are included.^27^

This study has some limitations. First of all, there were more tetraplegic than paraplegic patients in this study. Together with age, we included this variable as predictors in the URP-CTREE analyses to adjust for age and lesion level dependency. Furthermore, the clinical endpoints and some of the predictors used in this study represent ordinal variables. Even though it might be favorable to completely work with continuous variables, it has been shown that the URP-CTREE model is efficiently applicable to regression models where predictors and clinical endpoints are of nominal, ordinal, discrete, or continuous nature.^26^ Second, no midsagittal tissue bridges were observed in 1 patient graded AIS B and in 1 patient graded AIS D. Although this seems surprising, it might well be that potentially spared tracts run more laterally, which are not covered by segmentation of midsagittal tissue bridges. Furthermore, the low spatial resolution of T2w axial slices did not allow quantification of lateral tissue bridges. This limits the information about spared tracts and their relationships to specific functions. Nevertheless, we are confident that we captured the tracts mentioned in the discussion using midsagittal tissue bridges due to the slice thickness used for sagittal T2w images. Future studies could still benefit from optimizing axial T2w sequences to get information on more laterally located tracts. Finally, for part of the group-comparison and regression analyses, AIS grade as well as AIS conversion rate information from the acute stage was used. This allowed us to explore a larger subgroup of SCI patients improving in AIS grade over time. Furthermore, 12-month SCIM data were not available for all SCI patients. However, the dropout rate was relatively small with missing data from only 2 patients.

In conclusion, this study provides evidence that preserved midsagittal tissue bridges are able to predict AIS grade conversion after SCI. Using unbiased recursive partitioning analyses, we were furthermore able to identify ventral and dorsal tissue bridges in addition to conventional clinical measures as predictors splitting the patient cohort into more homogeneous subgroups regarding their specific recovery of motor score and pin-prick score as well as SCIM score, respectively. Preserved ventral and dorsal tissue bridges therefore add value in predicting neurological and functional recovery and consequently improve the accuracy of these prediction models. Prediction-based stratification analyses including neuroimaging biomarkers as predictors might help to further optimize identification of subgroups of responders in future clinical trials.
